# Proton-boron capture interaction enhances killing of radiation resistant cancer cells

**DOI:** 10.3389/fonc.2025.1720143

**Published:** 2025-12-11

**Authors:** Dalong Pang, Mira Jung, Alfredo Velena, William Parke, Anatoly Dritschilo

**Affiliations:** 1 Department of Radiation Medicine, Georgetown University Medical Center, Washington, WA, United States; 2 Department of Physics, George Washington University, Washington, WA, United States

**Keywords:** boron, proton, irradiation, cell survival, γH2AX, SQ-20B, MCF-7, BSH

## Abstract

**Background/objectives:**

Studies involving the interaction of protons with boron (^11^B) have shown potential for enhanced cell killing in cancer cells. However, theoretical analyses conducted using Monte Carlo simulations have not corroborated the experimental findings. Our objective is to independently investigate the effects of proton-boron capture interaction on the killing of cancer cells in SQ20-B and MCF-7 cells.

**Methods:**

Cell survival and DNA damage endpoints were analyzed in radiation resistant SQ-20B cells and in radiation sensitive MCF-7 cancer cells after exposure to ^11^B (BSH_11_) and proton irradiation. Clonogenic cell survival curves were assessed to fit the Linear Quadratic (LQ) and Single-Hit Multi-Target (SHMT) models. Additionally, γH2AX foci were quantified to evaluate DNA damage up to 24 hours post irradiation, comparing the effects of proton irradiation alone to proton irradiation in the presence of boron in SQ-20B cells.

**Results:**

Exposure of cells to BSH_11_ resulted in decreased survival of SQ-20B cells following proton irradiation as compared to untreated control cells. Assays measuring γH2AX showed prolonged presence of γH2AX foci in cells after proton exposure in the presence of BSH_11_. In contrast, cells treated with BSH_11_ and irradiated with Cs-137 γ-rays did not show cell killing enhancement. Additionally, cells treated with BSH_10_, an analog of BSH_11_ that contains only ^10^B, displayed no change in survival after proton irradiation compared to untreated cells.

**Conclusions:**

Our data show a small enhancement of cell killing by proton radiation in the presence of BSH_11_ that we attribute to the proton-boron interaction. Analysis of γH2AX demonstrates a prolonged duration of foci formation in cells after proton irradiation in the presence of BSH_11_. Further research will be needed to better understand the potential clinical applications of proton-boron interaction.

## Introduction

The primary advantage of proton radiotherapy (RT) compared to photon RT is the unique energy deposition associated with the Bragg peak, which results in no radiation dose delivered beyond this peak (1, 2). In addition to utilizing the inherent physical and biological properties of protons in radiotherapy, further strategies have been explored to enhance their biological effectiveness (3, 4).

One of the strategies is to explore interaction between proton and boron and the products it produces. Upon absorption of a proton, a stable isotope of boron (^11^B) is converted into a ^12^C nucleus in an excited state, which subsequently decays to an ^8^Be nucleus and emits a α particle of energy 3.76 MeV. The ^8^Be nucleus further decays into two α particles of energy 2.74 MeV each (5). When placed in a cell medium, the three α particles produced in this process possess enough energy to traverse several cancer cells of typical thickness of 10-20 µm. Mindful of the high lethality of α particles (6, 7), one can expect an enhancement of cell killing by proton irradiation when a ^11^B containing compound is introduced into the cell medium.

The experimental data presented by Cirrone et al. on the effectiveness of proton-boron capture therapy offered the first evidence of the potential efficacy of proton-boron capture therapy (PBCT) (8). However, subsequent theoretical studies have questioned the validity of the mechanisms proposed in this report (5, 9, 10). The concerns are primarily based on Monte Carlo simulation of the proton-boron capture interaction and the amount of energy released in the process, with a consensus among the authors that the energy released is too small to support the magnitude of effects on cell survival reported by Cirrone et al.

This study reports additional experimental data on potential efficacy of proton-boron interaction in cell killing. By employing a ^11^B containing compound (BSH_11_) and two cancer cell lines of different radiation sensitivity, we hope to gain insight on the potential cell killing effects of α particles as well as that of protons. To account for the fact that BSH_11_ contains 80% ^11^B and 20% ^10^B and the presence of secondary neutrons in the proton irradiation experiments, we irradiated with protons cells treated with BSH_10_ compound that contains over 99.97% ^10^B to evaluate the possibility of cell killing resulting from ^10^B and neutron capture interaction. In addition, BSH_11_ treated cells were also irradiated with photons to evaluate potential biological effects of BSH_11_.

## Materials and methods

Two cell lines were used in this study: the radioresistant SQ20-B cell line, which originated from squamous carcinoma of head and neck origin and is known for its radiation resistance and the radiosensitive MCF-7 cell line, which was derived from breast cancer and is more radiosensitive than other cell lines. These two cell lines allow for an analysis of the different radiation effects induced by densely ionizing particles, such as alpha particles which may have the potential to overcome the radiation resistance of cancer cells (6, 7). In addition to colony formation-based survival studies, we assessed DNA double-strand break (DSB) damage by quantifying γH2AX at various time points following irradiation.

### Cell growth and cytotoxicity assay

Cells were obtained from the Lombardi Cancer Center Tissue Culture Core Facility at Georgetown University Medical Center. SQ-20B cells were maintained in complete DMEM media: DMEM, 10% Fetal Bovine Serum (FBS), 5% Pen/Strep. MCF-7 cells were cultured in complete RPMI media: RPMI, 10% Fetal Bovine Serum (FBS), 5% Pen/Strep. Both cell lines were kept in logarithmic growth phase at 37^0^C and 5% CO_2_ environment and tested negative for mycoplasma contamination.

Cytotoxicity assays were performed by exposing cells to graded drug dilutions for 48 hours. Cytotoxicity was measured using the CellTox Green Cytotoxicity Assay (Cat.#G8731, Promega), following manufacturer’s instructions. Positive and negative controls were included to ensure proper normalization of the results. Cytotoxicity was assessed by measuring fluorescence at 513 nm excitation/532 nm emission. Cell viability is expressed as a percentage of the control groups. The inhibitory concentration values of 50% (IC_50_) values and the mean value of IC_50_ were calculated using Prism (GraphPad Software, Inc).

### Boron containing BSH compounds

This investigation utilized two BSH compounds: BSH_11_ and BSH_10_. The compounds, BSH_11_ (sodium mercaptododecaborate or N-BSH, Na_2_[1-SH^11^B_12_H_11_]) and BSH_10_ Compound (sodium mercaptododecaborate (10B), BSH, Na_2_[1-SH^10^B_12_H_11_] were purchased from KATCHEM Ltd, a commercial vendor (Czech Republic). The primary compound used in this research was BSH_11_, which has a boron isotope distribution of 80% ^11^B and 20% ^10^B_;_ in contrast, BSH_10_, which contains 99.97% ^11^B, served as a control to assess the potential effects of boron neutron capture reaction during interactions with BSH_11_ and protons. The BSH compounds were in powder form and were dissolved in deionized, distilled water to achieve appropriate dilutions, resulting in a concentration of 50 µM, equivalent to 6.6 ppm for B_11_.

### Proton irradiation

**Figure 1:**
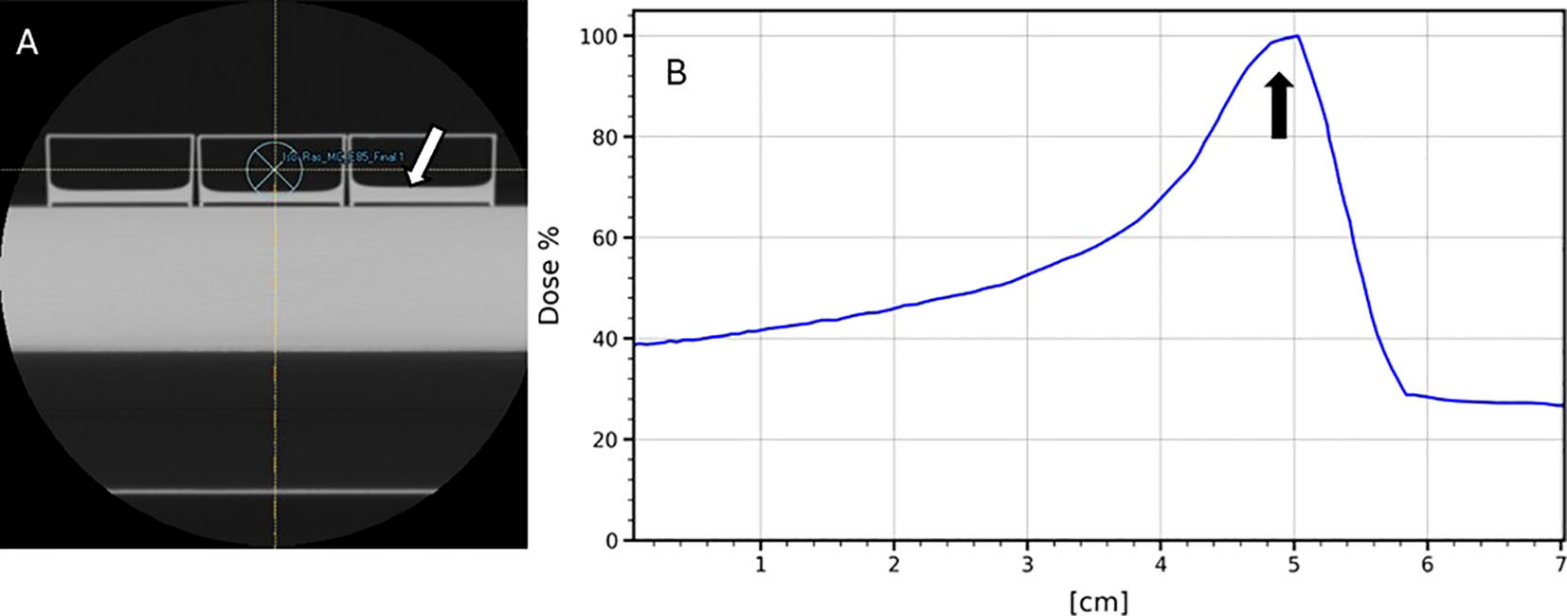
CT image of the irradiation apparatus **(A)** and the Bragg peak curve calculated by the Raystation treatment planning system. The arrow in **(A)** shows the location of the cell medium in one of the petri dishes, and the arrow in **(B)** shows the location of cell medium relative to the Bragg peak. The Y-axis shows the calculated RBE dose of proton along the beam path normalized to the maximum dose at the Bragg peak. The X axis shows the distance starting from the bottom of the solid water plate.

Proton irradiations were conducted at the Proton Center of Medstar Georgetown University Hospital using the Mevion S250i pencil beam scanning proton system. The radiation field consisted of a single energy layer of 85 MeV, with a field size of 20x20 cm^2^, comprising 1681 spots spaced 5 mm apart. Each spot received a monitor unit (MU) of 1.358. Cells in petri dishes, in a medium 1-mm thick, were positioned on top of a 5-cm solid water plate and CT scanned. **Figure 1A** shows the transverse CT image of the apparatus, where the light gray colored block shows the solid water plate and 3 containers on top show the petri dishes; the light gray colored layers at the bottom of the dishes show the cell containing liquid, as pointed by the white arrow. The CT images were imported into a Raystion treatment planning system (RS11A) by Raysearch Laboratories (Stochholm, Sweden) to generate radiation dose distribution from a single proton beam as defined above and directed posteriorly at the bottom of the solid water plate for irradiation of the cells. **Figure 1B** shows the Bragg peak curve of the beam and the arrow shows the position of the cells. This setup ensured that the dose delivery to the cell medium was maintained within the proximal 90% to distal 90% region of the Bragg peak. The doses were further measured using a calibrated parallel plate chamber with measurements taken in 1-mm increment along the beam direction to confirm that the dose variation within the irradiated medium is less than 10%. For each radiation delivery, six-well plates containing cells were placed within the treatment field. Doses of 0 (sham), 1, 3, 5, 7 and 9 Gy were administered in triplicate to facilitate statistical error determination.

### Photon irradiation

Photon irradiation of the cells was conducted at Georgetown University Lombardi Cancer Center**’**s core facility using a Cs-137 γ−irradiator on BSH_11_ treated SQ-20B cells. The dose rate of this irradiator was 1.56 Gy/min. Sample preparation and the doses delivered were consistent with previously detailed methods, except for the radiation source.

### Cell survival assay

Cells were seeded into T25 flasks and treated with the drug for 48 hours prior to proton irradiation. The treatments included a Sham group (no drug) and the BSH_11_ compound at a concentration of 50 μM. As a control to evaluate any potential cell-killing effects of neutron-boron interaction that might occur in the proton-BSH_11_ reaction, the BSH_10_ compound was infused in another batch of SQ-20B cells at the same concentration. Each treatment was conducted in triplicate. Following irradiation, cells were incubated for 10–14 days post radiation until colony formation was observed. The cell survival data were fitted with the Linear-Quadratic (LQ) model for calculation of survival at various dose levels and alpha and beta values, The Single-Hit-Multi-Target (SHMT) model was also used for determination of D_0_. Mean and standard deviation were calculated from three repeats of each experiment. Unpaired student t-test was used for evaluation of statistical significance.

### H2AX analysis

SQ-20B cells were treated with the BSH_11_ compound at a concentration of 50 μM for 48 hours prior to exposure to 5 Gy of proton radiation. Sham-treated cells served as control. Cells were fixed for 15 min using 4% formaldehyde at various time points (30min, 1hr, 2hrs, 4hrs, 6hrs, 12hrs and 24hrs) following irradiation. After fixation in 4% Formaldehyde, the cells were blocked for 1 hour using a Blocking Buffer (95% PBS, 5% Goat Serum and 0.3% TritonX-100) and incubated with γH2AX antibody (Abcam) overnight at 4^0^C, followed by incubation with a secondary anti-rabbit Alexa Fluor 488 for 1hr. Fluorescence images were captured using a Leica SP8 AOBS Laser Scanning Confocal Microscope after mounting the cells with Scale bar = 200 µm for all images. The number of distinct foci in individual nuclei was counted for each condition. Two-sample unpaired t-tests were performed for statistical analysis.

## Results

There were no observed cytotoxicities to BSH11 or BSH10 exposures of SQ-20B or MCF7 cells to concentration from 1 to 50 μM. BSH is non-toxic in these cancer cells at the concentrations used in these experiments. Therefore, we selected a concentration of 50 μM for radiation experiments.

**Figure 2:**
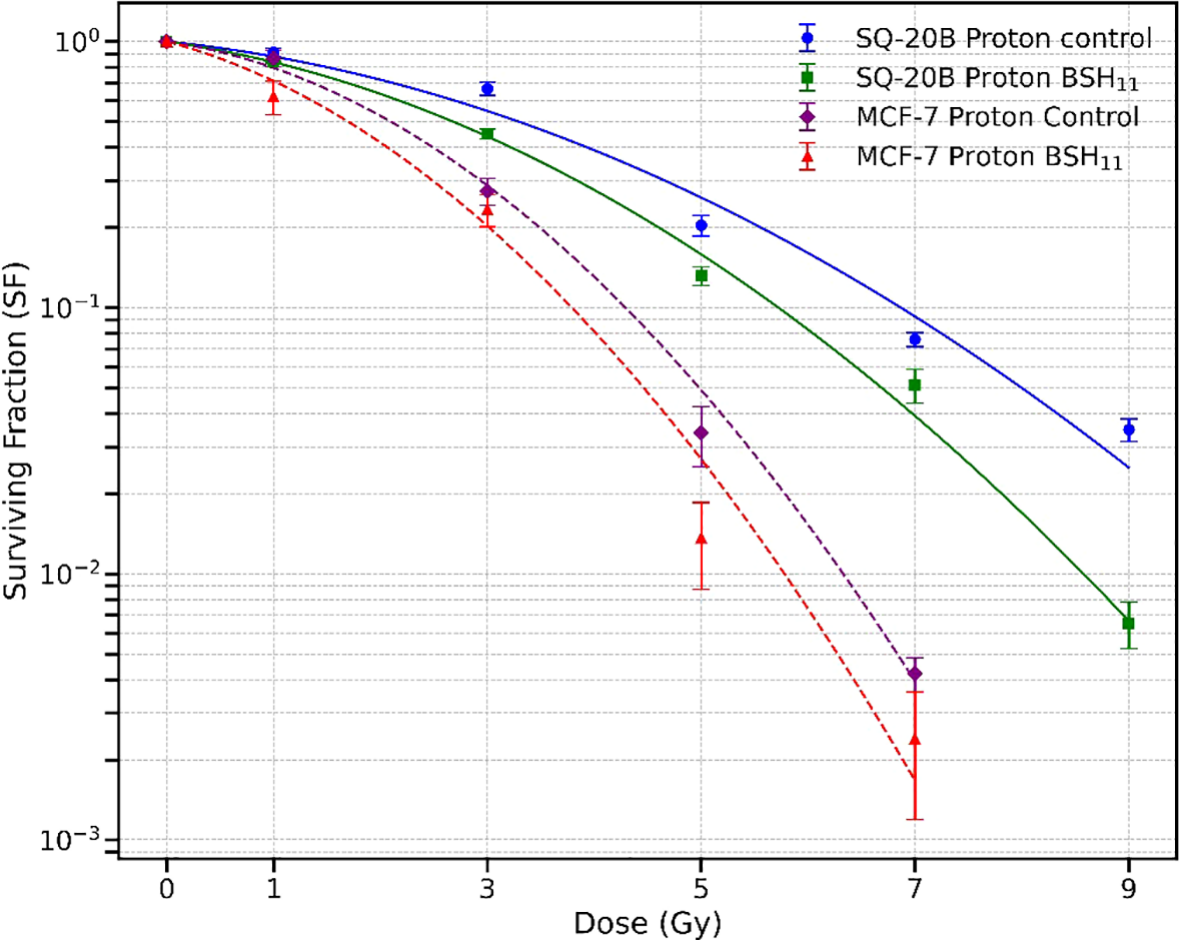
Survival curves for SQ-20B (solid lines) and MCF-7 (dashed lines) cell lines following proton irradiation, with and without 50 µM BSH_11_. For SQ-20B, circles represent control (no BSH_11_) and squares represent BSH_11_-treated cells; for MCF-7, diamonds represent control, and triangles represent BSH_11_-treated cells. Data points represent the mean ± standard error from three independent experiments. Curves were obtained from log-scale least-squares fit the experimental data.

**Figure 2** shows the cell survival curves for SQ20-B and MCF-7 cells irradiated with protons alone and with protons combined with 50 µM BSH_11_. Solid curves correspond to SQ-20B, while dashed curves correspond to MCF-7. Across the dose range, the addition of BSH_11_ reduced survival for both cell lines, consistent with a radiation sensitizing effect. Radiation resistant SQ-20B cells consistently exhibited higher survivals than MCF-7 cells.

Dose by dose analysis using the LQ model revealed that BSH_11_ treatment significantly decreased SQ-20B survival at 3 Gy (p = 0.0069), 5 Gy (p = 0.0266), 7 Gy (p = 0.0473), and 9 Gy (p = 0.0014) indicating a pronounced effect at intermediate and high doses. For MCF-7, although survival was consistently lower in the BSH_11_ group, the dose points did not reach statistical significance (p > 0.05). The LQ model derived D_10_ (dose required to reduce cell survival to 10%) further reflected these differences. In SQ-20B cells, D_10_ decreased from 6.890 ± 0.134 Gy to 5.776 ± 0.080 Gy with BSH_11_ (p = 0.0002), corresponding to a dose-modifying factor (DMF - ratio of the dose required to achieve 10% survival without BSH to that with BSH) of 1.19, which indicates an enhanced overall radiation sensitizing effect. In MCF-7 cells, D_10_ decreased from 4.322 ± 0.090 Gy to 3.994 ± 0.512 Gy (p = 0.3360), giving a DMF of 1.08, but this change was not statistically significant.

From the SHMT model, the D_0_, defined as the dose that reduces cell survival to 37% in the exponential portion of the survival curve, was significantly reduced in SQ-20B cells from 1.874 ± 0.108 Gy (control) to 1.523 ± 0.102 Gy with BSH_11_ treatment (p = 0.0149). This decrease indicates an increased intrinsic radiosensitivity of SQ-20B cells in the presence of BSH_11_. In contrast, MCF-7 cells showed no significant changes in D_0_ between control (0.941 ± 0.105 Gy) and BSH_11_-treated (0.948 ± 0.154 Gy) groups (p = 0.9529) which suggests that the intrinsic sensitivity of MCF-7 to proton irradiation was not altered by BSH_11_.

**Figure 3:**
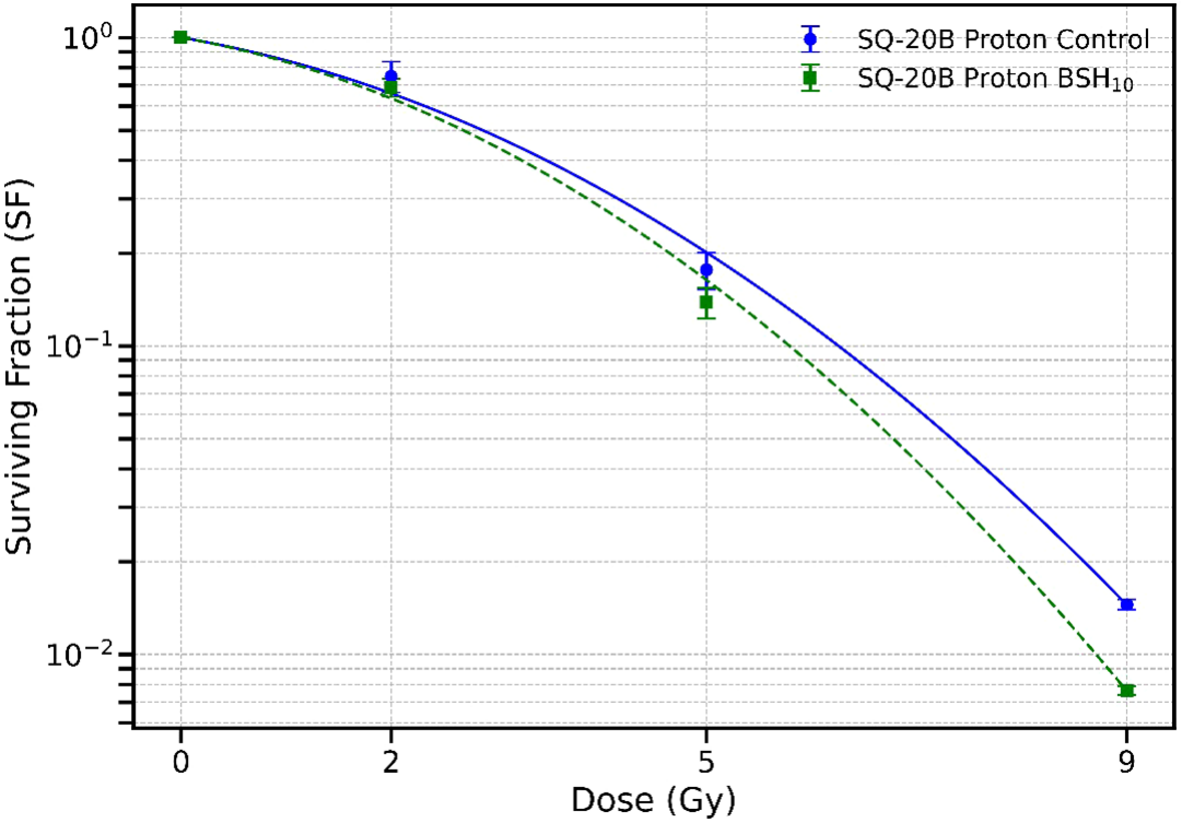
Survival curves for SQ-20B cells irradiated with protons alone (circles) or with 50 µM BSH_10_ treatment (squares). Data points represent the mean ± standard error from three independent experiments. Curves were fitted using the Linear–Quadratic (LQ) model. No statistically significant differences in D_10_ were observed between the two groups (p = 0.1117).

The BSH_11_ compound contains 80% ^11^B and 20% ^10^B; therefore, the ^10^B component could contribute to the observed cellular effects of BSH_11_ through the boron-neutron interaction. To investigate this possibility, we irradiated SQ-20B cells with protons after treatment with BSH_10_ which contains 99.97% of ^10^B. **Figure 3** shows the resulting survival curves. No statistically significant differences in survival were observed between the BSH_10_-treated and control groups with D_10_ values of 6.23 ± 0.30 Gy and 5.78 ± 0.23 Gy, respectively (p = 0.1117). These findings suggest that ^10^B, in the absence of ^11^B, does not significantly alter proton radiosensitivity in SQ-20B cells under the tested conditions.

**Figure 4:**
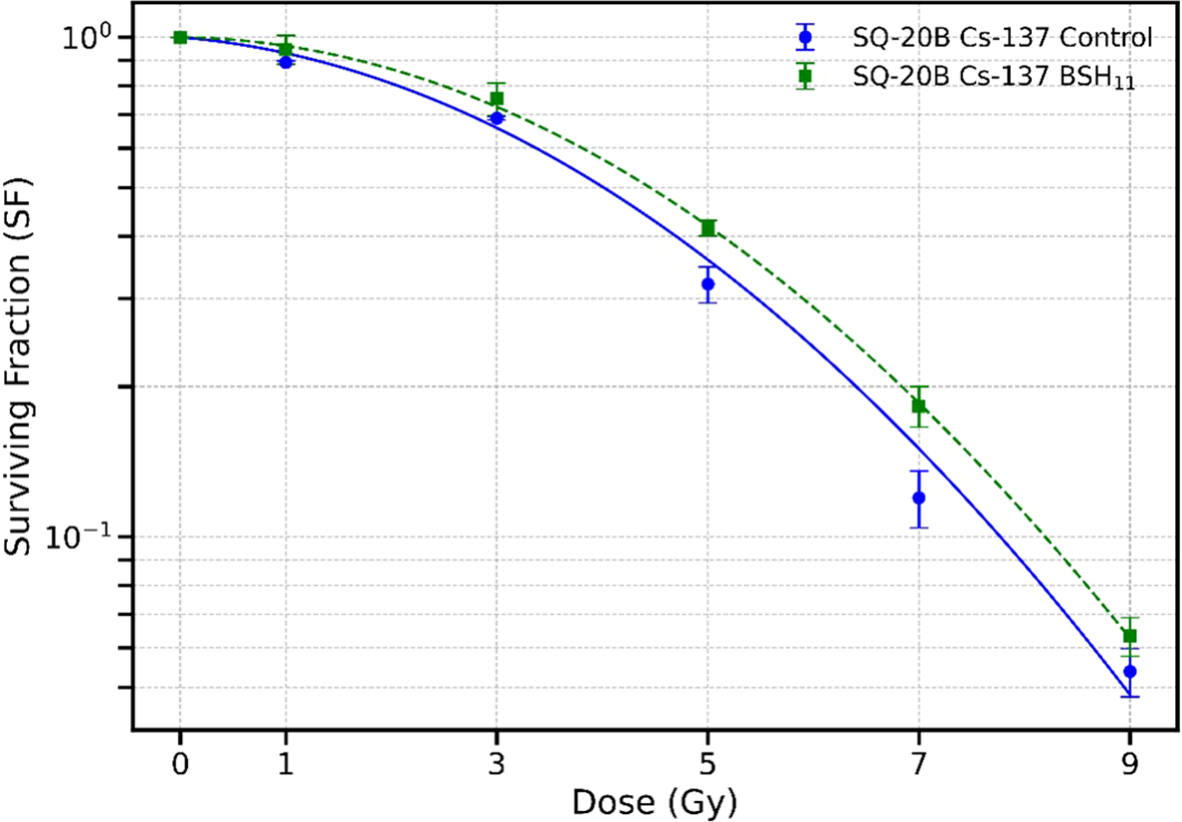
Survival curves for SQ-20B cells irradiated with Cs-137 gamma rays in the absence (circle) and presence (square) of 450 µM BSH_11_. Data points represent the mean ± standard error from three independent experiments. Data fitted with LQ model. No statistically significant difference in survival was observed between the two groups with D_10_ values of 7.78 ± 0.23 Gy (control) and 8.21 ± 0.25 Gy (BSH_11_) (p = 0.0920).

To further evaluate the role of the proton-^11^B interaction, we irradiated SQ-20B cells with Cs-137 gamma rays in a Cesium irradiator with and without BSH_11_ treatment. To rule out any purely chemical effects of the BSH compound, the BSH_11_ concentration was increased from 50 to 450 µM. **Figure 4** shows the corresponding survival curves. Treatment with BSH_11_ did not produce a statistically significant change in survival when cells were irradiated with gamma photons, with D_10_ values of 7.78 ± 0.23 Gy for control cells and 8.21 ± 0.25 Gy for BSH_11_-treated cells (p = 0.0920). These results indicate that the survival differences observed in proton-irradiated SQ-20B cells are unlikely to be due to chemical effects of BSH_11_ but rather to the specific interaction between protons and ^11^B.

### H2AX

γH2AX is a highly sensitive molecular marker for detecting the presence of DNA double strand breaks and the subsequent repair of these lesions (11). To investigate whether the observed reduction in cell survival in the presence of BSH_11_ may be the result of a possible change in the DNA DSB pattern, potentially attributable to proton-boron capture interaction, we irradiated the SQ-20B cells with a single fraction, proton dose of 5 Gy, both with and without BSH. We conducted γH2AX analyses on the irradiated cells up to two hours post irradiation.

**Figure 5:**
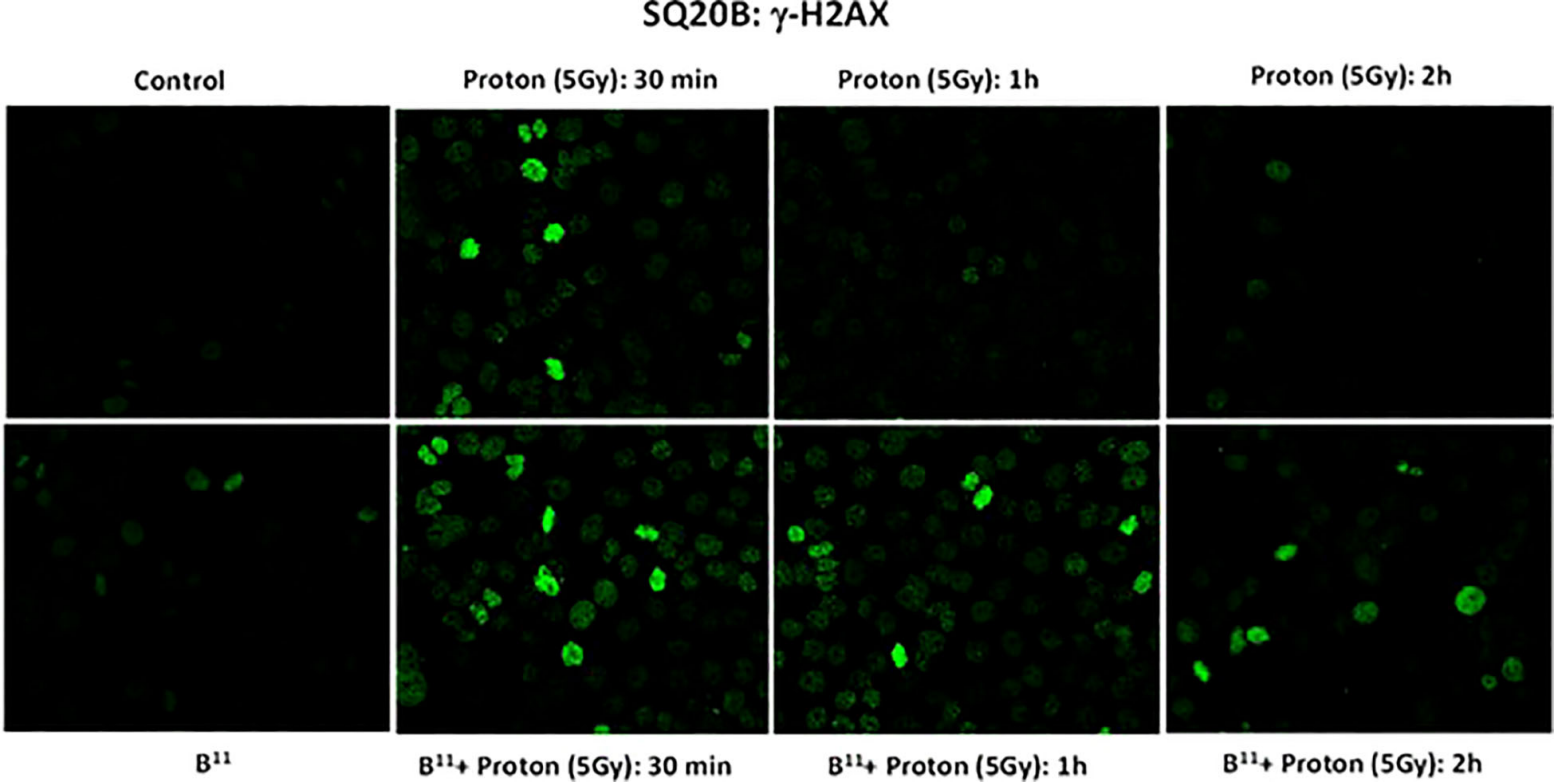
γH2AX foci formation for SQ-20B cells post irradiation at 30 mins, 1 hr and 2 hr time intervals.

As shown in **Figures 5**, the number of foci per cell was significantly higher in BSH_11_ treated SQ-20B cells and persisted longer following exposure to proton irradiation (5Gy) compared to irradiated cells without BSH_11_ treatment (all P < 0.001).

**Figure 6:**
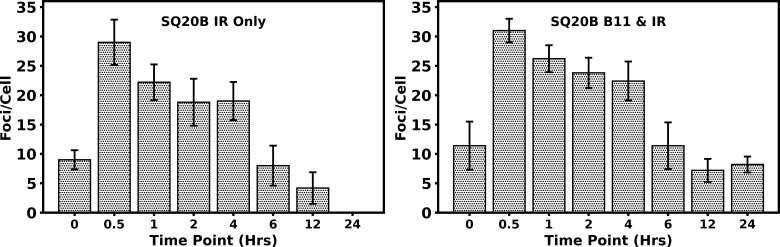
Analysis of γH2AX foci in SQ-20B (B) cells. Following treatment at various time points, the number of cells with foci formation was counted in three different fields. Each field contained as average 50–100 cells, which were examined to determine the number of foci formations. The 0-hour samples were treated with BSH_11_ only or subjected to sham irradiation. Error bars represent the standard error, and the percentage of focus-positive cells (cells containing at least 10 foci) is plotted. The histogram was created using the average number of foci in five cells within each field at different time points.

We further evaluated the kinetics of the repair processes by assessing foci formation at various time points, ranging from 0 hours to 24 hours, across three different fields. Each field contained an average of 50 to 100 cells, which were analyzed to determine the number of foci formed. **Figure 6** shows the foci numbers peaking at 30 minutes and then decreasing over a 6-hour interval. The proton + BSH_11_ treated cells demonstrate a slower recovery process.

The difference in the number of foci per cell in BSH_11_ treated cells after radiation—compared to cells without BSH_11_ treatment—suggests that nearly all double-strand breaks (DSBs) induced by proton radiation were repaired within 24 hours in SQ-20B cells. In contrast, treatment with BSH_11_ altered this response in two important ways: it increased the number of breaks at the same radiation dose and slowed their repair.

## Discussion

Investigations of the effectiveness of proton-boron interaction have produced inconsistent results, ranging from radiation killing enhancement to no apparent effects (8, 12–17). Our experiments comparing two different cancer cell lines, one radioresistant and the other sensitive, reveal a small sensitization of the radiation resistant cells, but not of the radiation sensitive cells. As illustrated in **Figure 2**, the addition of BSH_11_ to the SQ-20B cells led to statistically significant reduction in survival across all dose levels except at the lowest dose of 1 Gy. For the MCF-7 cells, although the survival appears to be lower in the presence of BSH_11_ at all dose levels, the reductions are not statistically significant.

We interpret these observations to support a radiation sensitizing effect of BSH_11_ on radiation resistant cells and suggest that the alpha particles released in the p-^11^B interaction may contribute to the observed increase in cell killing of the SQ-20B cells. This interpretation is consistent with the observations of high-LET radiation overcoming radiation resistance. For the more radiation sensitive MCF-7 cells, sensitization was observed (**Figure 2**) but did not reach statistical significance in our experiments. Nonetheless, the sensitization effect on radiation resistant cell lines is the focus of this research.

In a therapeutic proton system, neutrons are also generated due to scattering interactions with various atoms along the beam path. For the Mevion S250i proton system, the neutron contamination in the primary beam is less than 0.1% (18). Despite this low neutron flux, it can still damage certain electronics in the treatment room, potentially including cells in the petri dish used for experiments. Recent Monte Carlo simulations by other investigators have estimated the presence of high energy and thermal neutrons in proton irradiated materials, including cell medium (19, 20) and found a thermal neutron flux on the order of 10^8^/cm^2^/GyE and potential radiation sensitization due to BNCT effects by irradiating cells of various cell lines treated with BPA (19). A small sensitization was reported at BPA concentration of 80 ppm which was attributed to the BNCT effect (19), suggesting the possibility of thermal neutron contribution in PBCT due to the 20% ^10^B contained in BSH_11_. In this research we investigated the potential role of ^10^B by irradiating cells treated with BSH_10_ which contains over 99.97% ^10^B with no ^11^B and found no sensitization and thus ruled out the BNCT effects due to effects of possible thermal neutrons under our experimental conditions.

The results obtained in the p-BSH_11_, p-BSH_10_ and γ-BSH_11_ experiments support the interpretation that the reduced cell survival in the p-BSH_11_ experiment was likely due to the interaction between proton and ^11^B which produces three alpha particles. These alpha particles are lethal in cell killing when traversing cells and may induce additional bystander cell killing effects (21, 22).

It is interesting to observe that BSH_11_ induced a statistically significant survival reduction in the radiation resistant SQ-20B cells but not the radiation sensitive MCF-7 cells. Further experiments will be needed to assess the effects of higher concentrations.

## Conclusions

The experimental data on cell survival that we present in this report for SQ-20B and MCF-7 cells, following proton and photon irradiation in the presence of BSH, demonstrate a small but statistically significant increase in cell death in the radiation resistant SQ-20B cells but not the radiation sensitive MCF-7 cells. Our findings are less robust but qualitatively support Cirrone’s report of enhanced cell kill by protons in the presence of ^11^B. This enhancement can be attributed to the boron-proton interaction under our experimental conditions. The potential clinical relevance may require further studies.

## Data Availability

The raw data supporting the conclusions of this article will be made available by the authors, without undue reservation.
